# Lnc SMAD5-AS1 as ceRNA inhibit proliferation of diffuse large B cell lymphoma via Wnt/β-catenin pathway by sponging miR-135b-5p to elevate expression of APC

**DOI:** 10.1038/s41419-019-1479-3

**Published:** 2019-03-15

**Authors:** Chen-Chen Zhao, Yang Jiao, Yi-Yin Zhang, Jie Ning, Yi-Ruo Zhang, Jing Xu, Wei Wei, Gu Kang-Sheng

**Affiliations:** Department of Oncology, The First Affiliated Hospital of Anhui Medical University, Hefei, Anhui Province China

## Abstract

Diffuse large B cell lymphoma (DLBCL) is a common and fatal hematological malignancy. Long noncoding RNAs (lncRNAs) have emerged as crucial biomarkers and regulators in many cancers. Novel lncRNA biomarker in DLBCL needs to be investigated badly, as well as its function and molecular mechanism. To further explore, microarray analysis was performed to identify the differentially expressed lncRNAs in DLBCL tissues. To investigate the biological functions of SMAD5-AS1, we performed gain- and loss-of-function experiments in vitro and in vivo. Furthermore, bioinformatics analysis, dual-luciferase reporter assays, Argonaute 2-RNA immunoprecipitation (AGO2-RIP), RNA pull-down assay, quantitative PCR arrays, western blot assay, TOPFlash/FOPFlash reporter assay, and rescue experiments were conducted to explore the underlying mechanisms of competitive endogenous RNAs (ceRNAs). We found that SMAD5-AS1 was down-regulated in DLBCL tissues and cell lines. Functionally, SMAD5-AS1 downregulation promoted cell proliferation in vitro and in vivo, whereas SMAD5-AS1 overexpression could lead to the opposite effects in vitro and in vivo. Bioinformatics analysis and luciferase assays revealed that miR-135b-5p was a direct target of SMAD5-AS1, which was validated by dual-luciferase reporter assays, AGO2-RIP, RNA pull-down assay, and rescue experiments. Also, dual-luciferase reporter assays and rescue experiments demonstrated that miR-135b-5p targeted the adenomatous polyposis coli (APC) gene directly. SMAD5-AS1/miR-135b-5p inhibits the cell proliferation via inactivating the classic Wnt/β-catenin pathway in the form of APC dependency. Our results indicated that SMAD5-AS1 inhibits DLBCL proliferation by sponging miR-135b-5p to up-regulate APC expression and inactivate classic Wnt/β-catenin pathway, suggesting that SMAD5-AS1 may act as a potential biomarker and therapeutic target for DLBCL.

## Background

Diffuse large B cell lymphoma (DLBCL) is a kind of non-Hodgkin’s lymphoma, which accounts for about 25–35% in non-Hodgkin’s lymphoma and 37% in B cell tumor in the world^1^. DLBCL is a highly aggressive diffuse malignant hyperplastic disease of the lymphatic system, and clinical therapeutic regimens used currently are ineffective in about 40% patients^2^. The reason for it is that there is a lack of obvious symptoms in the early stage of DLBCL and its pathogenesis remains unclear, so no effective targeted therapy has been found, leading to poor prognosis and low 5-year survival rate of only 40%^3^. According to the cell of origin (COO), DLBCL is divided into several subtypes, providing a certain basis for clinical treatment and prognosis^4^. According to differential expression of B cell development-related genes, DLBCL can be divided into at least four subtypes^5^, mainly including activated B cell (ABC) lymphoma, germinal center B cell (GCB) lymphoma, primary mediastinal B cell lymphoma and unclassified subtype. Rituximab, cyclophosphamide, doxorubicin, vincristine, and prednisolone widely used in the clinical treatment of DLBCL have good therapeutic effects on ABC lymphoma, but have poor effects on other subtypes^6^. In addition to understanding DLBCL from COO and selecting clinical drugs, it is also very important in oncobiology to find the original mutant gene leading to DLBCL. The chromosomal translocation caused by Myc, Bcl-2, and/or Bcl-6 structural reorganization is closely related to the therapeutic effect and prognosis of disease. However, the gene mutation varies from person to person, and it is even different in different cells in the same tumor, so no good therapeutic effect has been obtained in the signal molecule in COO typing or the targeted therapy for the original mutant gene products. Therefore, there is an urgent need to find new targeted therapeutic molecules for DLBCL.

Long noncoding ribonucleic acid (LncRNA) was first discovered in the mouse cDNA (complementary DNA) library by Japanese scientists Okaznki et al^7^. LncRNA was once considered as the “junk sequence” and “transcriptional noise,” because it does not encode the protein. Until 2007, Rinn et al.^8^ found lncRNA-HOTAIR with 2.2 kb in length and confirmed that it can inhibit the HOX gene transcription through mobilizing the protein complex Polycomb, thus regulating the growth and development of organism. Since then increasingly more attention has been paid to the identification and functional study on lncRNA. LncRNA plays an important role in the occurrence, development, invasion, and metastasis of tumor, which is considered as an emerging biomarker and potential therapeutic target in the epigenetics of cancer^9^. For example, H19 can promote the oncogenicity, invasion, and angiogenesis of glioblastoma;^10^ EWSAT1 (Ewing sarcoma-associated transcript 1)-mediated gene regulation promotes the occurrence of Ewing’s sarcoma^11^, and the reduced expression of growth arrest-specific transcript 5 (GAS5) can promote the occurrence of non-small cell lung cancer (NSCLC)^12^. LncRNA related to the occurrence and development of DLBCL was found in this study using the gene expression profiling screening technique, and its function and regulatory mechanism were identified, so as to provide new ideas for enriching the pathogenesis of DLBCL and guidance of clinical treatment.

## Methods

### Tissue samples

Resected DLBCL lymph gland and adjacent normal lymph gland biopsies were collected from The First Affiliated Hospital of Anhui Medical University from January 2013 to January 2015. There were 11 DLBCL samples and 11 normal adjacent samples. All tissues were directly stored in liquid nitrogen at −80 °C. Informed consent was obtained from each participant. The use of human clinical tissues was approved by the Institutional Human Experiment and Ethics Committee of The First Affiliated Hospital of Anhui Medical University. The Declaration of Helsinki was strictly followed during experiments.

### Microarray profiling

TRIzol reagent (Invitrogen, Carlsbad, CA, USA) was used to extract total RNA, which was then purified by a RNeasy Mini Kit (Qiagen, Valencia, CA, USA). Differentially expressed lncRNAs in DLBCL tissues were screened by the whole-genome microarray expression profiling based on the criteria of log 2 (fold change) >1.5 and adjusted *P* **<** 0.05. The manufacturer’s standard protocols were strictly followed. Briefly, cDNA was synthesized, labeled, and purified. lncRNA microarray chips were hybridized by Cyanine-3-CTP-labeled cRNA. After washing, samples were analyzed on the lncRNA microarray. The differentially expressed genes were calculated and clustered by R program.

### Cell line culture

Cell lines were purchased from American Type Culture Collection (ATCC, Manassas, VA, USA), including GCB DLBCL cell lines (TMD8 and U2932), human B lymphocyte (GM12878), and human embryonic kidney cell line (HEK-293). ABC DLBCL cell line (OCI-Ly3), follicular lymphoma cell line (WSU-FSCCL), mantle cell lymphoma cell line (JeKo-1), classic Hodgkin's lymphoma cell line (L428), and Burkitt's lymphoma cell line (Raji) were purchased from the cell bank of Chinese Academy of Sciences. Cells were maintained in modified RPMI-1640 medium, supplemented with 10% fetal bovine serum, including 100 μg/L penicillin and 100 μg/L streptomycin. All cell lines were grown with 5% CO_2_ at 37 °C.

### Real-time quantitative polymerase chain reaction

TRIzol reagent (Invitrogen, Carlsbad, CA, USA) was used to isolate total RNA from tissues and cells according to the manufacturer’s instructions. Moloney Leukemia Virus Reverse Transcriptase Kit (Promega, Madison, WI, USA) was then performed to reverse transcribe total RNA (1 μg) to cDNA. Target primers were amplified by SYBR Green Mix (Promega). Sequences of the primers are listed in Table 1. All primers were synthesized by Shanghai Tingzhou Biological Engineering Co., Ltd. The miR-135b-5p level was performed using the TaqMan MicroRNA Assay Kit (Applied Biosystems, Carlsbad, CA, USA) according to the manufacturer’s instructions. All results were calculated and expressed as 2^−ΔΔCt^. Glyceraldehyde 3-phosphate dehydrogenase (GAPDH) was used as an endogenous control for SMAD5-AS1 and APC and U6 for miR-135b-5p. Triplicate is required for each experiment.

**Table 1:** Sequences used in this study

ID	Sequences
SMAD5-AS1 forward	5′-GAGCACCGCAGTCCTATCAA-3′
SMAD5-AS1 reverse	5′-GCTGGGTGACTCCTACCATC-3′
APC forward	5′-AGGGTGTCACTGGAGACAGA-3′
APC reverse	5′-TCTTCAGTGCCTCAACTTGCT-3′
GAPDH forward	5′-TGAACGGGAAGCTCACTGG-3′
GAPDH reverse	5′-TCCACCACCCTGTTGCTGTA-3′
U6 forward	5′-CTCGCTTCGGCAGCACA-3′
U6 reverse	5′-AACGCTTCACGAATTTGCGT-3′
SMAD5-AS1 shRNA	Top strand: 5′-CACCGCTGTCCAATGGCTTGATGAATCCGTTCAAGAGACGGATTCATCAAGCCATTGGACAG-3′;
	Bottom strand: 5′-AAAACTGTCCAATGGCTTGATGAATCCGTCTCTTGAACGGATTCATCAAGCCATTGGACAGC-3′)

*APC* adenomatous polyposis coli, *GAPDH* glyceraldehyde 3-phosphate dehydrogenase, *shRNA* short hairpin RNA

### RNA isolation of nuclear and cytoplasmic fractions

The Nuclear/Cytoplasmic Isolation Kit (Biovision) was applied to isolate and collect cytosolic and nuclear fractions. RNA levels of SMAD5-AS1, RNU6-1 (nuclear control transcript), and GAPDH (cytoplasmic control transcript) were analyzed by real-time quantitative polymerase chain reaction (RT-qPCR).

### In situ hybridization

Cells were seeded onto poly-l-lysine-treated glass slides for 24 h after trypsinization harvest and then fixed in methanol at −20 °C for 5 min. The in situ hybridization (ISH) assays were performed as previously described^13^. A locked nucleic acid (LNA) probe with complementarity to a section of SMAD5-AS1 (5′-GGCCGGTCGCCGACTTATACCACTT-3′ custom LNA detection probe, Exiqon) was labeled with digoxigenin (DIG) antibody (Roche, 11,093,274, 1:1000) and synthesized. The intensity and the extent of staining were evaluated by two pathologists who were blinded to the experiment.

### Fluorescence in situ hybridization

TMD8 and U2932 cells were fixed in 4% paraformaldehyde for 15 min. Then, 0.5% Triton X-100 was used to permeabilize the cells for 15 min at 4 °C. DIG-labeled SMAD5-AS1 probe or control probe mix was performed to incubate cells for 4 h at 55 °C. After a brief wash with 2 ×  saline-sodium citrate for 5 min (5–6 times), the signal was detected by horseradish peroxidase-conjugated anti-DIG secondary antibodies (Jackson, West Grove, PA, USA). Olympus confocal laser scanning microscope was applied for obtaining the image. 4′,6-Diamidino-2-phenylindole was used as a nuclear counterstain.

### Lentivirus production and cell transfection

The pBLLV-CMV-IRES-ZsGreen SMAD5-AS1 cDNA lentiviral plasmid and lentivirus-containing short hairpin RNA (shRNA) targeting SMAD5-AS1 plasmid were purchased from Genelily BioTech Co., Ltd, (Shanghai, China). The cells were selected by puromycin (2 μg/mL) for 2 weeks at 48 h after transfection. Cell lines with stable SMAD5-AS1 silence or overexpression was then constructed. RT-qPCR was performed to verify the transfection efficiency. The miR-135b-5p mimic, miR-135b-5p inhibitor, and negative control (NC) oligonucleotides were obtained from Tingzhou Biological Engineering Co., Ltd (Shanghai, China). The above-mentioned oligonucleotides and plasmids were transfected by using Lipofectamine 3000 (Invitrogen). The manufacturer’s instructions were strictly followed.

### Cell Counting Kit-8 assay

Cells (2 × 10^4^ cells/ml) were seeded onto 96-well plates (100 μl per well) and then placed in an incubator with 5% CO_2_ at 37 °C for 24 h. After the cells were cultured for 5 days, 10 μl of Cell Counting Kit-8 (CCK8) solution was added to each well. The absorbance values at a wavelength of 450 nm were measured to evaluate cell viability.

### MTT assay

Transfected DLBCL cells seeding in a 96-well plate (1 × 10^4^ cells per well) were incubated for 24, 48, 72, and 96 h. MTT (3-[4,5-dimethylthiazol-2-yl]-2,5 diphenyl tetrazolium bromide; 10 μl of 5 mg/mL) was added to each well to incubate for another 4 h. Then, dimethyl sulfoxide (Thermo Fisher Scientific, Waltham, MA, USA) was added (100 μl per well) after removing the supernatants. A microplate reader was used to measure the absorbance value at a wavelength of 490 nm.

### Flow cytometry

The flow cytometry assays were performed as previously described^14^. Apoptosis was determined with the Annexin V-FITC early apoptosis kit and a flow cytometer. DLBCL cells transfected with SMAD5-AS1 overexpression plasmid or shSMAD5-AS1 or NC were analyzed on the flow cytometer (FACScan; BD Biosciences) and were calculated using the CellQuest software (BD Biosciences).

### Western blot analysis

Western blot analyses were performed according to standard protocols as previously described^15^. Anti-catenin-β, anti-APC, and anti-lamin B1 were purchased from Sigma.

### Luciferase reporter and TOPFlash/FOPFlash reporter assays

The reporter vector pmirGLO-SMAD5-AS1-wt (wild type) was formed by cloning SMAD5-AS1 cDNA, which contains the predictive binding site of miR-135b-5p, into the pmirGLO Dual-Luciferase miRNA Target Expression Vector (Promega). The vector pmirGLO-SMAD5-AS1-Mut (mutant) was inserted by the mutant SMAD5-AS1, which contains point mutations of the miR-135b-5p seed region binding site. HEK-293FT cells were cultured and co-transfected with pmirGLO-SMAD5-AS1–3′-UTR (untranslated region) vectors including wild-type or mutant fragments, miR-135b-5p and miR-NC. Likewise, wild-type and mutant APC 3′-UTR fragments were cloned into the pmirGLO vector. The miR-135b-5p or miR-NC was co-transfected with APC-wt or APC-Mut vector into HEK-293FT cells using Lipofectamine 3000 (Invitrogen). The Dual-Luciferase Reporter Assay System (Promega) was applied at 48 h after transfection according to the manufacturer’s instructions. For the TOPFlash/FOPFlash reporter assay, Wnt/β-catenin signaling reporter TOPFlash/FOPFlash (Addgene, Cambridge, MA, USA) was co-transfected into cells along with SMAD5-AS1 silence or overexpression vector, miR-135b-5p mimic or inhibitor, and/or the microRNA (miRNA) control. Normalized TOPFlash/FOPFlash values were presented as previously described^16^. Experiments were performed in triplicate.

### RNA immunoprecipitation

The EZMagna RIP Kit (Millipore) was applied according to the manufacturer’s protocol. Complete RNA immunoprecipitation (RIP) lysis buffer was used to lyse DLBCL cells. Magnetic beads conjugated with anti-Argonaute 2 (AGO2) or control anti-immunoglobulin G (IgG) antibody were used to incubate the cell extract. The cell extract was incubated for 6 h at 4 °C. Then, as the protein beads were removed RT-qPCR analysis was conducted for the purification of RNA.

### RNA pull-down assay

Briefly, TMD8 cells were transfected with the 3′ end biotinylated miR-135b or miR-135b-mut for 24 h at a final concentration of 20 nmol/L. Then, the cells were incubated in the cell lysate with streptavidin-coated magnetic beads (Ambion, Life Technologies). The biotin-coupled RNA complex was pulled down and analysis of the abundance of SMAD5-AS1 in bound fractions was then conducted by RT-qPCR. The pull-down assay was performed as previously described^17^.

### Xenograft tumor model

Xenograft tumor model was performed in BALB/c-nude mice (4–5 weeks of age), which were purchased from Shanghai SLAC Laboratory Animal Co., Ltd, China. The experimental procedures were approved by the Institutional Animal Care and Use Committee of our institution. Tumor growth was monitored every 5 days; tumor volumes were estimated by length and width. One month later, the mice were sacrificed, and then tumors were excised and weighed.

### Statistical analysis

SPSS 22.0 statistical software package and GraphPad Prism 7.0 were applied for statistical analyses.

All data are represented as mean ± standard deviation. To compare two or more groups, the Student’s *t* test or one-way analysis of variance were performed for different analysis. Differences were considered statistically significant when *P* < 0.05.

## Results

### LncRNA-SMAD5-AS1 was down-regulated in DLBCL tissues and cell lines

**Fig. 1:**
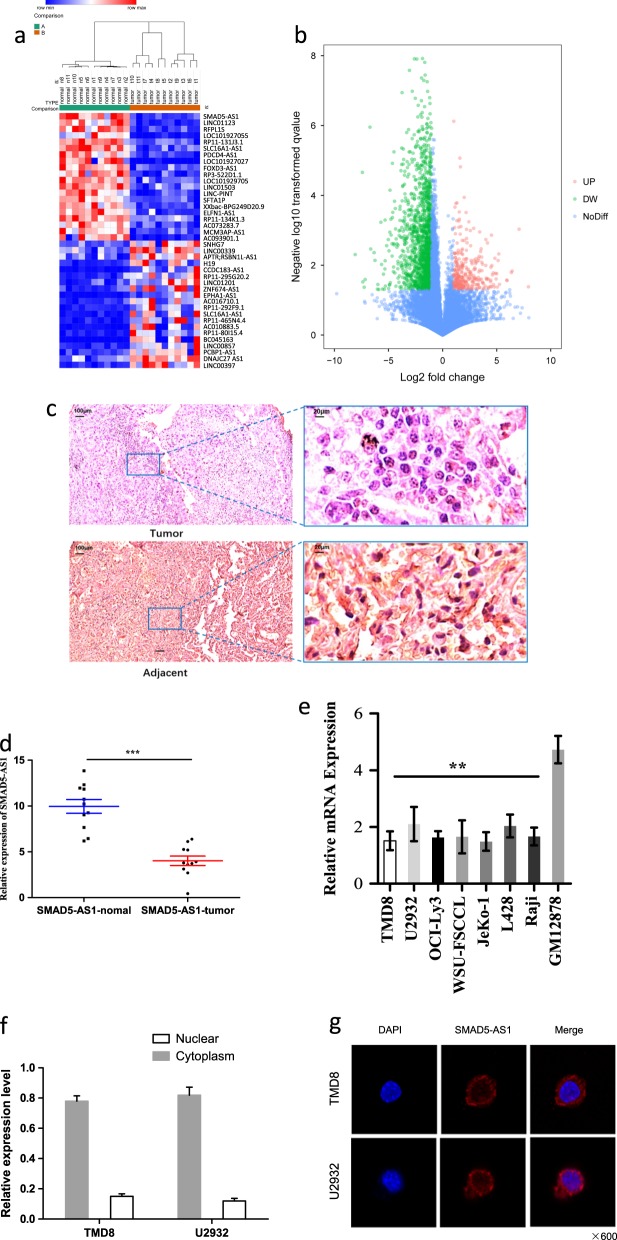
Long noncoding RNA (LncRNA) SMAD5-AS1 is down-regulated in diffuse large B cell lymphoma (DLBCL) tissues and cell lines. **a** The heat map with hierarchical cluster of the top 40 differentially expressed lncRNAs between DLBCL samples and normal samples (>1.5-fold; *P* < 0.05). **b** LncRNAs with fold change >1.5 and *P* < 0.05 were plotted in the volcano plot. A volcano plot is constructed by plotting the negative log of the *P* value on the *y*-axis. These result in data points with low *P* values appearing toward the top of the plot. The *x*-axis is the log of the fold change between the DLBCL and normal samples. **c** In situ hybridization detection of SMAD5-AS1 in DLBCL tissue and adjacent normal tissue (×100 and ×400). **d** Real-time quantitative polymerase chain reaction (RT-qPCR) was performed to validate SMAD5-AS1 expression in DLBCL samples and normal samples (*n* = 11 vs. 11). ***P < 0.001. The expression of SMAD5-AS1 was lower in tumor tissues than that in normal tissues. **e** RT-qPCR was performed to measure the relative expression of SMAD5-AS1 in several lymphoma cell lines and normal human B lymph cells (GM12878). The majority of SMAD5-AS1 was located in the cytoplasm according to the nuclear mass separation assay (**f**) and fluorescence in situ hybridization (**g**)

A total of 40 lncRNAs (fold change >1.5, *P*_adj_ <0.01) differentially expressed in DLBCL tissues were screened (Fig. 1a, b). As shown in the heat map, SMAD5-AS1 was significantly down-regulated in tumor tissues compared with that in normal tissues (Fig. 1a). LncRNA-SMAD5-AS1 was named as it is an antisense transcript of gene SMAD5 according to Human Gene Nomenclature Committee. It localized in chromosome 5: 136,129,507–136,134,890 reverse strand with 2 exons. Zavadil et al.^18^ have firstly identified it in fetal and several tumor tissues. The function and regulation of this LncRNA remained unclear as few studies focused on it. The results of ISH and RT-qPCR revealed that the expression of SMAD5-AS1 was lower in tumor tissues than that in normal tissues (Fig. 1c, d). According to the results of RT-qPCR, the expression of SMAD5-AS1 in lymphoma cell lines was significantly reduced compared with that in normal human B lymphocyte cell line (GM12878) (Fig. 1e), and there was a statistically significant difference (*P* < 0.05). The differences among lymphomas were not statistically significant. The subcellular localization of SMAD5-AS1 in DLBCL cell lines was determined using the nuclear mass separation assay and fluorescence in situ hybridization (FISH), and it was found that SMAD5-AS1 was mainly located in the cytoplasm (Fig. 1f, g).

### Up-regulation of SMAD5-AS1 inhibited cell viability and cycle and promoted apoptosis

**Fig. 2:**
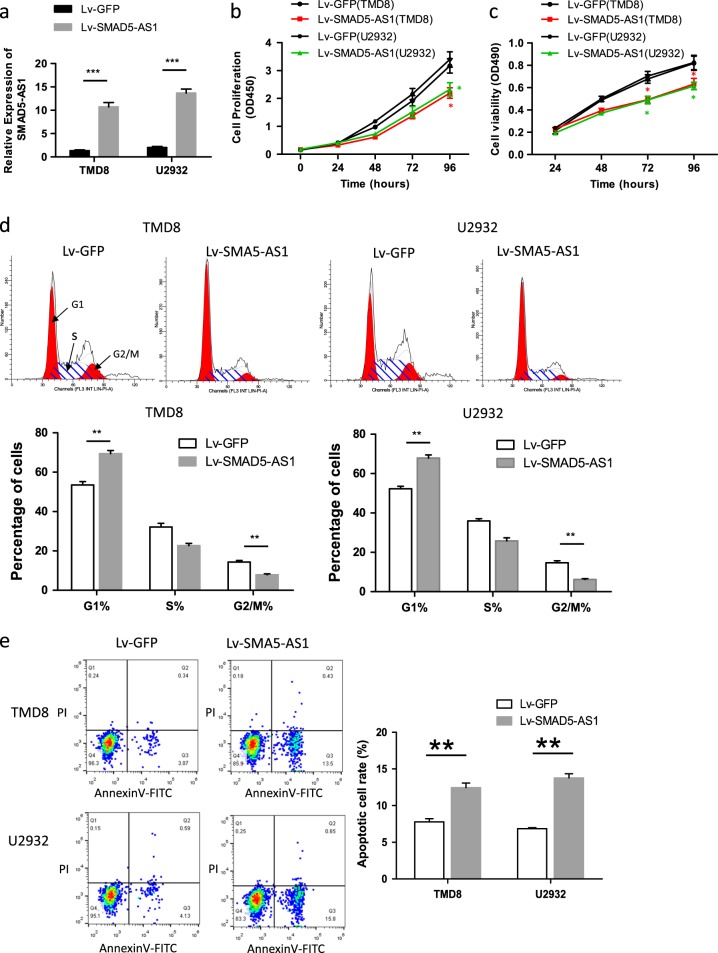
SMAD5-AS1 inhibits cell proliferation of diffuse large B cell lymphoma (DLBCL) cells in vitro. **a** Efficiency of SMAD5-AS1 expression in SMAD5-AS1-overexpressed TMD8 and U2932 cells was evaluated by real-time quantitative polymerase chain reaction (RT-qPCR). The expression of SMAD5-AS1 in SMAD5-AS1-overexpressed group was obviously increased compared with the control group. ***P < 0.001. Cell proliferation and viability were measured by Cell Counting Kit-8 (CCK8) (**b**) and MTT (3-[4,5-dimethylthiazol-2-yl]-2,5 diphenyl tetrazolium bromide) (**c**) assays. The cell proliferation and viability were reduced in SMAD5-AS1-overexpressed group compared with that in the control group. *P < 0.01. **d** The phase of the cell cycle was detected by fluorescence-activated cell sorting (FACS) analysis. Pictures of flow cytometry were shown in the upper panel. (The area under the left red peak chart represents the proportion of cells in the G1 phase. The area under the right red peak chart represents the proportion of cells in the G2/M phase. The area of oblique line represents the proportion of cells in the S phase.), while comparison was performed in the corresponding histograms of lower panels. The number of cells in the G1/G0 phase was increased in the SMAD5-AS1-overexpressed group, while that in the G2/M phase was declined. **P < 0.01. **e** Annexin V assay was used to determine cell apoptosis rate. Overexpression of SMAD5-AS1 promoted apoptosis of lymphoma cells. **P < 0.01. *N* = 3 independent experiments

After transfection with the vector containing SMAD5-AS1-overexpressed plasmid, the expression of SMAD5-AS1 in TMD8 and U2932 cells was obviously increased compared with that in empty vector group (Fig. 2a), the cell proliferation and viability were reduced compared with that in empty vector group (Fig. 2b, c) according to the results in CCK8 and MTT assays, the cell cycle was inhibited compared with that in empty vector group (Fig. 2d), and the apoptotic rate was increased compared with that in the empty vector group (Fig. 2e). The results displayed a statistically significant difference (*P* < 0.05).

### Down-regulation of SMAD5-AS1 enhanced cell viability, promoted cell cycle, and inhibited apoptosis

**Fig. 3:**
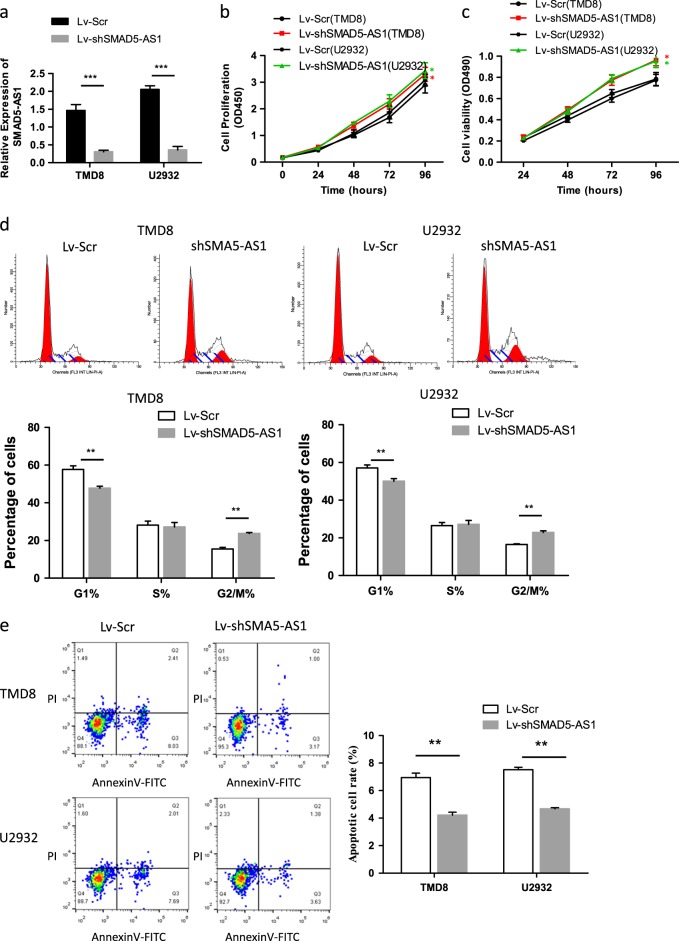
Down-regulated SMAD5-AS1 promotes cell proliferation of diffuse large B cell lymphoma (DLBCL) cells in vitro. **a** Efficiency of SMAD5-AS1 expression in SMAD5-AS1 down-regulated TMD8 and U2932 cells was evaluated by real-time quantitative polymerase chain reaction (RT-qPCR). The expression of SMAD5-AS1 in shSMAD5-AS1 group was obviously decreased compared with control group. ***P < 0.001. Cell proliferation and viability were measured by Cell Counting Kit-8 (CCK8) (**b**) and MTT (**c**) assays. The cell proliferation and viability were enhanced in shSMAD5-AS1 group compared with that in control group. *P < 0.01. **d** The phase of the cell cycle was detected by FACS analysis. Pictures of flow cytometry were showed in the upper panel, while comparison was performed in the corresponding histograms of lower panels. The number of cells in the G1/G0 phase was decreased in shSMAD5-AS1 group, while that in the G2/M phase was elevated **P < 0.01. **e** Annexin V assay was used to determine cell apoptosis rate. Down-regulation of SMAD5-AS1 inhibited apoptosis of lymphoma cells **P < 0.01. *N* = 3 independent experiments

After transfection with the vector containing shSMAD5-AS1 plasmid, the expression of SMAD5-AS1 in TMD8 and U2932 cells was obviously reduced compared with that in the empty vector group (Fig. 3a), the cell proliferation and viability were increased compared with that in the empty vector group (Fig. 3b, c) according to the results in CCK8 and MTT assays, the cell cycle was promoted compared with that in the empty vector group (Fig. 3d), and the apoptotic rate was inhibited compared with that in the empty vector group (Fig. 3e). The results showed a statistically significant difference (*P* < 0.05). It is remarkable that 3′ exonic sequences of SMAD5-AS1 contain, in part, an alternate 5′ exon of SMAD5 in the antisense orientation^18^. To avoid that observed phenotype was due to the alteration of SMAD5, we further validated the effect of alteration of SMAD5-AS1 on SMAD5. The result indicated that the expression of SMAD5 was completely not affected by alteration of SMAD5-AS1, though possible binding sites existed (data not shown). It could be confirmed that the expression of SMAD5 gene altered negligibly in this study as the expression of SMAD5-AS1 was exogenously intervened.

### In vivo verification of influence of SMAD5-AS1 on cell proliferation ability

**Fig. 4:**
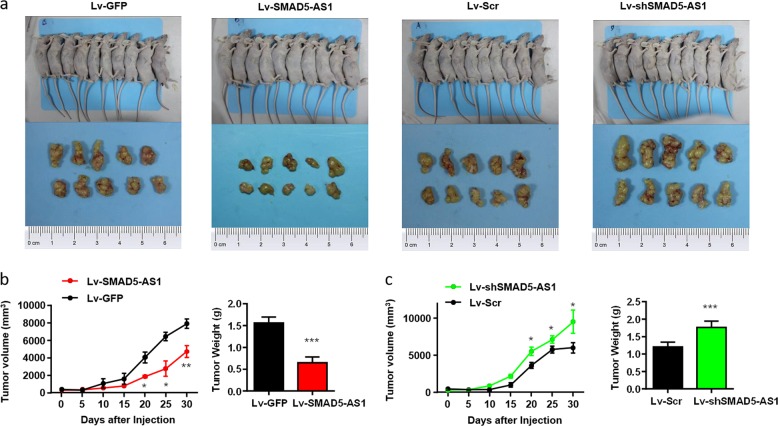
SMAD5-AS1 inhibits proliferation in vivo. **a** The nude mice were injected with TMD8 cells transfected with SMAD5-AS1-overexpressed plasmid/control plasmid or shSMAD5-AS1 plasmid/control plasmid subcutaneously, which was shown in the upper panel. Tumors were removed after 1 month, which were indicated in the bottom panel. Tumor volume and tumor weight were analyzed in SMAD5-AS1-overexpressed/control group (**b**) and shSMAD5-AS1/control group (**c**). The transplanted tumor with the overexpression of SMAD5-AS1 had a smaller volume and lower weight and the transplanted tumor with the down-regulation of SMAD5-AS1 had a larger volume and higher weight. *P < 0.05

To evaluate the influence of SMAD5-AS1 on proliferation ability, nude mice were injected subcutaneously with the TMD8 cells with stable overexpression and down-regulation of SMAD5-AS1 and corresponding control cells. As expected, the transplanted tumor with the overexpression of SMAD5-AS1 had a smaller volume and lower weight than that in control group (Fig. 4a), while the transplanted tumor with the down-regulation of SMAD5-AS1 had a larger volume and higher weight than that in the control group (Fig. 4b), and the differences were statistically significant (*P* < 0.05).

### SMAD5-AS1 played a role in DLBCL as an endogenous competitive RNA of miR-135b-5p and regulated target gene APC of miR-135b-5p

**Fig. 5:**
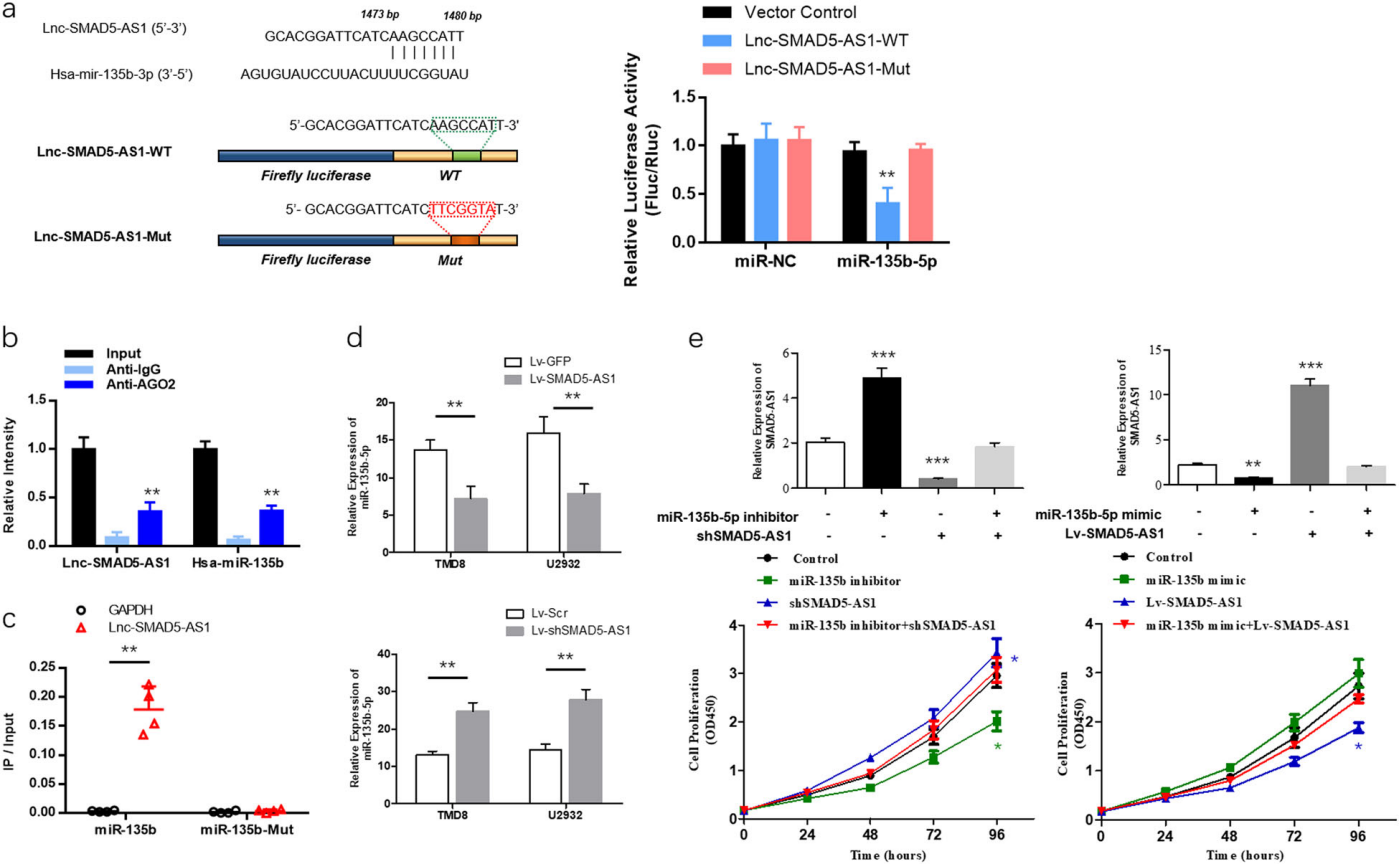
SMAD5-AS1 served as a molecular sponge for miR-135b-5p. **a** Schematic diagram showed the putative miR-135b-5p binding sites with the SMAD5-AS1. The sequences of wild-type SMAD5-AS1 and mutant SMAD5-AS1 were listed as well. Luciferase reporter gene assays were performed to measure the luciferase activity in TMD8 cells. **P < 0.01. **b** Anti-Argonaute 2 (AGO2) RNA immunoprecipitation (RIP) assays were used in TMD8 cells to determine SMAD5-AS1 and miR-135b-5p RNA enrichment in immunoprecipitated (IP) complex. Anti-immunoglobulin G (IgG) was used as the control. SMAD5-AS1 and miR-135b-5p were enriched preferentially in miRNA ribonucleoprotein complexes (miRNPs)^1–3^ containing AGO2 compared with anti-IgG immunoprecipitates. **P *<* 0.01 **c** The biotinylated miR-135b or its mutant (miR-135b-mut) was transfected into TMD8 cells. Real-time quantitative polymerase chain reaction (RT-qPCR) was applied to quantify the RNA levels of SMAD5-AS1 and glyceraldehyde 3-phosphate dehydrogenase (GAPDH). Scatter plot showed the relative ratios of the input of IP. SMAD5-AS1 was more enriched in the wild-type miR-135b-5p compared with that in the mutant-type miR-135b-5p with broken SMAD5-AS1 binding site **P < 0.01. **d** The relative expression of miR-135-5p in diffuse large B cell lymphoma (DLBCL) cells transfected with SMAD5-AS1-overexpressed plasmid/control plasmid and shSMAD5-AS1 plasmid/control plasmid. **e** RT-qPCR and Cell Counting Kit-8 (CCK8) assay were applied to measure the relative expression of SMAD5-AS1 and cell proliferation in TMD8 cells at 36 h after transfection with control, miR-135b mimic or miR-135b inhibitor, SMAD5-AS1 overexpression plasmid or shSMAD5-AS1 plasmid, and miR-135b mimic + Lv-SMAD5-AS1 or miR-135b inhibitor + shSMAD5-AS1. *P < 0.05. *N* = 3 independent experiments

ceRNA is a well-known regulatory mechanism of lncRNA. LncRNA sponges a variety of miRNAs to inhibit its expression and reduce the regulatory effect on target messenger RNA (mRNA). The target recognition sequence of SMAD5-AS1 miRNA was analyzed via bioinformatics (RegRNA 2.0 http://regrna2.mbc.nctu.edu.tw/detection.html; Lncrnadb http://www.lncrnadb.org; miRcode http://www.mircode.org/), and it was found that miR-135b-5p had a complementary sequence to SMAD5-AS1. To prove the finding, SMAD5-AS1 cDNA was cloned into the luciferase gene (pGL3-PVT1-214-wt) and co-transfected with miR-135b-5p or miR-NC. The results revealed that the luciferase activity in the miR-135b-5p group was significantly reduced compared with that in miR-NC group. At the same time, the miR-135b-5p binding site was mutated, and the pGL3-PVT1-214-mut vector was produced. The results showed that the vector after mutation had no significant influence on the luciferase activity in the miR-135-5p group (Fig. 5a). RNA-induced silencing complexes (RISCs) are formed by miRNA ribonucleoprotein complexes (miRNPs), which is present in anti-Ago2 immunoprecipitates. Therefore, anti-Ago2 immunoprecipitates contain miRNAs and their interacting RNA components^19–21^. RIP assay was performed using anti-AGO2 in the TMD8 extract, and it was found that SMAD5 and miR-135b-5p were enriched preferentially in miRNPs containing AGO2 compared with anti-IgG immunoprecipitates (Fig. 5b). The results of RNA pull-down assay manifested that SMAD5-AS1 was more enriched in the wild-type miR-135b-5p compared with that in the mutant-type miR-135b-5p with broken SMAD5-AS1 binding site (Fig. 5c). The RT-qPCR results showed that the overexpression of SMAD5-AS1 in DLBCL cells could lead to the down-regulation of miR-135b-5p expression, while the down-regulation of SMAD5-AS1 could lead to the increase in miR-135b-5p expression (Fig. 5d). In the rescue experiment, the down-regulation of SMAD5-AS1 expression in TMD8 cells could reverse the increase in SMAD5-AS1 expression and inhibition on cell proliferation caused by the miR-135-5p inhibitor. Similarly, the overexpression of SMAD5-AS1 could reverse the loss of SMAD5-AS1 expression and enhancement of cell proliferation caused by the miR-135-5p mimic (Fig. 5e).

**Fig. 6:**
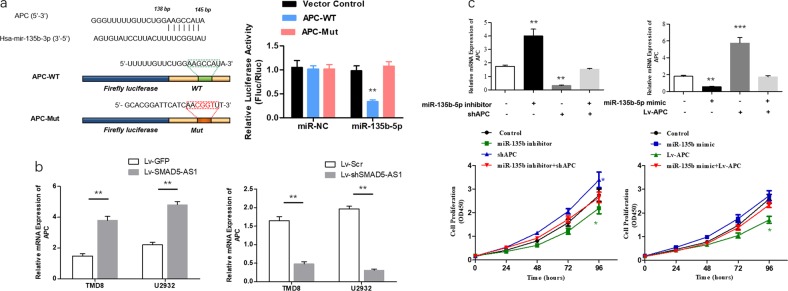
Adenomatous polyposis coli (APC) is identified as a direct target of miR-135b-5p in diffuse large B cell lymphoma (DLBCL) proliferation. **a** Schematic diagram showed the predicted miR-135b-5p binding sites with the 3′-untranslated region (UTR) of APC. The sequences of wild-type and mutant 3′-UTR of APC were listed as well. Luciferase reporter gene assays were performed to measure the luciferase activity in TMD8 cells. **P *<* 0.01. **b** The relative expression of APC in DLBCL cells transfected with SMAD5-AS1-overexpressed plasmid/control plasmid and shSMAD5-AS1 plasmid/control plasmid. **c** Real-time quantitative polymerase chain reaction (RT-qPCR) and Cell Counting Kit-8 (CCK8) assay were applied to measure the relative expression of APC and cell proliferation in TMD8 cells at 36 h after transfection with control, miR-135b mimic or miR-135b inhibitor, APC overexpression plasmid or shAPC plasmid, and miR-135b mimic + Lv-APC or miR-135b inhibitor + shAPC. *P < 0.05. *N* = 3 independent experiments

According to the prediction via bioinformatics (Targetscan 7.2 http://www.targetscan.org/vert_72/; miRDB http://www.mirdb.org/; miRTarBase http://mirtarbase.mbc.nctu.edu.tw/php/index.php), there might be a binding site in the APC 3′-UTR with miR-135-5p. Then, the luciferase assay confirmed that the 3′-UTR of wild-type APC could significantly lower the luciferase activity in miR-135b-5p group without significant influence on the luciferase activity in the miR-NC group. The 3′-UTR of mutant-type APC had no obvious influence on the luciferase activity in the miR-135b-5p group (Fig. 6a). Whether APC could be regulated by the expression of SMAD5-AS1 was verified then. According to the RT-qPCR results, the overexpression or down-regulation of SMAD5-AS1 could increase or decrease the mRNA expression in the APC gene (Fig. 6b). Moreover, the co-transfection of miR-135-5p mimic and APC-overexpressed plasmid or miR-135b-5p inhibitor and shAPC plasmid into TMD8 cells could restore the APC gene expression and cell proliferation (Fig. 6c).

### SMAD5-AS1/miR-135b-5p/APC axis regulated cell proliferation via Wnt/β-catenin signaling pathway

**Fig. 7:**
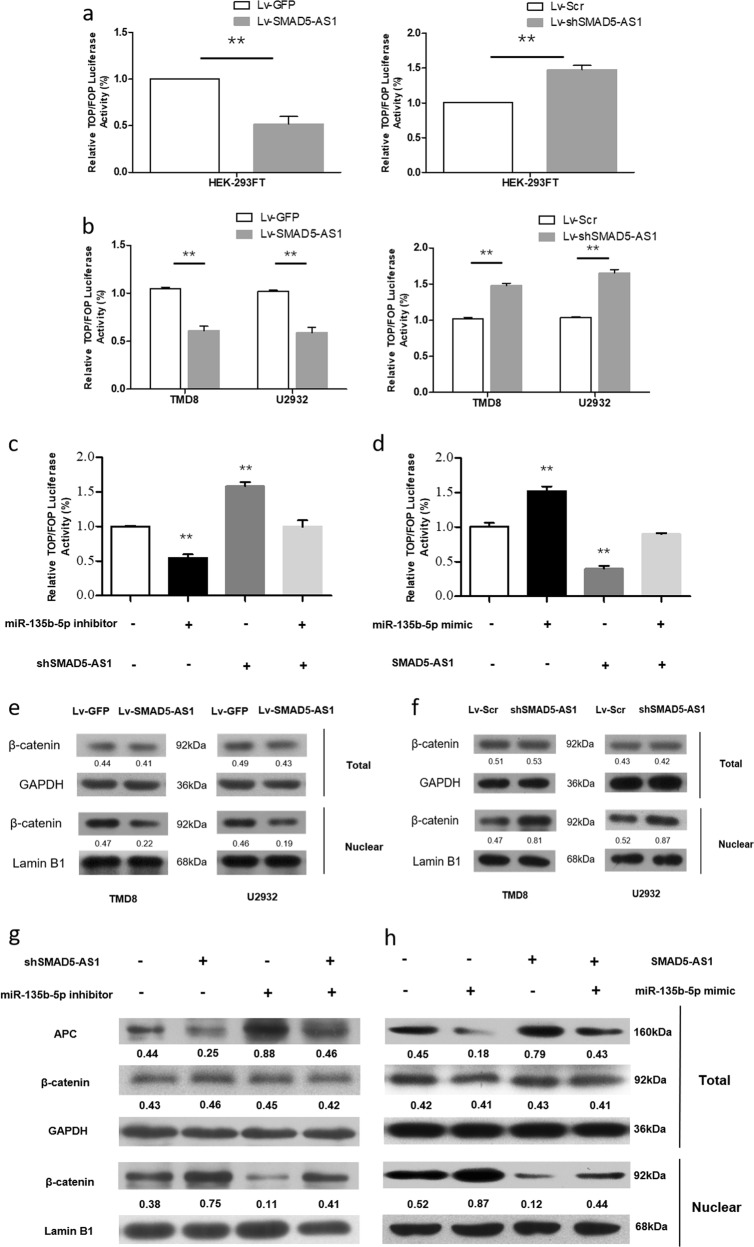
SMAD5-AS1 attenuates the proliferation ability of diffuse large B cell lymphoma (DLBCL) cells via adenomatous polyposis coli (APC)/Wnt/β-catenin pathway. **a**, **b** The effect on TOP/FOP reporter activity in HEK-293FT cells, and TMD8 and U2932 cells transfected with SMAD5-AS1 overexpression vector or shSMAD5-AS1 vector was proved by dual-luciferase assay. A Renilla transfection control normalized all results. **P < 0.01. **c**, **d** The effect on TOP/FOP reporter activity in TMD8 cells after transfection with control, miR-135b mimic or miR-135b inhibitor, SMAD5-AS1 overexpression plasmid or shSMAD5-AS1 plasmid, and miR-135b mimic + Lv-SMAD5-AS1 or miR-135b inhibitor + shSMAD5-AS1 was validated by dual-luciferase assay. **e**, **f** Western blot assay of total and nuclear β-catenin proteins in TMD8 and U2932 cells transfected with SMAD5-AS1-overexpressed plasmid or shSMAD5-AS1 plasmid and their corresponding control plasmid. Quantification of relative protein amount was shown by the number. Glyceraldehyde 3-phosphate dehydrogenase (GAPDH) and Lamin B1 were used as an internal control and an endogenous control of cell nuclear fraction, respectively. **g**, **h** Western blot assay of APC, total and nuclear β-catenin proteins in TMD8 cells transfection with control, miR-135b mimic or miR-135b inhibitor, SMAD5-AS1 overexpression plasmid or shSMAD5-AS1 plasmid, and miR-135b mimic + Lv-SMAD5-AS1 or miR-135b inhibitor + shSMAD5-AS1. Quantification of relative protein amount was shown by the number. GAPDH and Lamin B1 was used as an internal control and an endogenous control of cell nuclear fraction, respectively. *N* = 3 independent experiments

As a well-known gene related to the Wnt/β-catenin signaling pathway, the APC protein is closely related to the stability of β-catenin in the Wnt signaling pathway^22–24^.Therefore, the TOPFlash and FOPFlash reporters containing the consistent binding sites of wild-type and mutant-type T cell transcription factor 4 (TCF-4) were constructed to verify whether SMAD5-AS1/miR-135b-5p could regulate the classical Wnt pathway and affect the cell proliferation through regulating APC. The transcriptional activity of TOP/FOP was significantly reduced or enhanced in HEK-293FT cells with stable overexpression or down-regulation of SMAD5-AS1 (Fig. 7a). The similar results were also observed in TMD8 and U2932 cells (Fig. 7b). The down-regulation of SMAD5-AS1 expression in TMD8 cells could reverse the decline in the transcriptional activity of TOP/FOP caused by miR-135b-5p inhibitor (Fig. 7c), while the up-regulation of SMAD5-AS1 expression could reverse the enhancement of the transcriptional activity of TOP/FOP caused by miR-135b-5p mimic (Fig. 7d). Whether SMAD5-AS1 could affect the activation of Wnt/β-catenin pathway was also determined via western blotting, and the results manifested that the up- or down-regulation of SMAD5-AS1 could obviously modulate the expression of β-catenin in the nucleus (Fig. 7e, f). The results of rescue experiments by western blotting showed that the up-regulation of APC expression by miR-135b-5p inhibitor could be restored by the co-transfection of shSMAD5-AS1. The co-transfection of miR-135b-5p inhibitor and shSMAD5-AS1 failed to affect the total expression level of β-catenin. In the case of down-regulation of SMAD5-AS1, the expression of β-catenin in the nucleus was significantly increased. The nucleus/cytoplasm ratio of β-catenin expression in TMD8 cells transfected with shSMAD5-AS1 was far higher than that in TMD8 cells transfected with miR-135b-5p inhibitor, but such a difference was obviously narrowed by the co-transfection of them (Fig. 7g). The similar results were also observed in TMD8 cells transfected with miR-135b-5p mimic and/or SMAD5-AS1-overexpressed plasmid (Fig. 7h). The above results indicate that SMAD5-AS1 reduces the accumulation of β-catenin in the nucleus and the activation of classical Wnt pathway through inhibiting miR-135b-5p and promoting APC expression. The SMAD5-AS1/miR-135b-5p/APC axis regulated the cell proliferation via regulating classical Wnt pathway.

## Discussion

LncRNAs related to DLBCL have been studied in recent years. Peng et al.^25^ detected several DLBCL patients receiving no treatment and found that HULC is up-regulated in tumor tissues, and its expression is closely related to the characteristics of DLBCL patients, such as the Ann Arbor stage, B symptoms (systemic symptoms), and international prognostic index) score. Such a pro-cancerous effect of HULC may be related to the regulation of cyclin D1 and Bcl-2. Peng et al.^26^ proved via RT-qPCR that the high expression of paternally expressed 10 (PEG10) has a positive correlation with the progression of DLBCL, and the functional experiments proved that PEG10 promotes the cell proliferation and inhibits the apoptosis. Oh et al.^27^ found the close correlations among PRC2 family proteins (EZH2, SUZ12, and EED), H3K27me3, c-MYC, Bcl-2, and Hox transcript antisense intergenic RNA (HOTAIR) in DLBCL tissues, and two possible pathways were proposed: (1) HOTAIR may induce the effect of H3K27me3 via mobilizing most inhibitor complexes, EZH2 methyltransferase and core proteins SUZ12 and EED; (2) the tumor gene c-MYC may induce the effect of H3K27me3 through the PRC2-related pathway in DLBCL. Yan et al.^28^ knocked out HOTAIR in the in vitro experiment and found that the cell cycle is arrested in the G2/M phase, and the apoptosis increases. In the functional experiment, it was found that HOTAIR could inhibit the phosphorylation of P13K, AKT, and NF-κB, and it is believed that HOTAIR may inhibit the cell proliferation through inhibiting the P13K/AKT/NF-κB pathway. Li et al.^29^ found that the expression of MALAT-1 in DLBCL cell lines is significantly higher than that in normal human B lymphocytes. Besides, the survival rate of lymphoma cells declines after MALAT-1 knockdown in drug-resistant cell lines, the proportion of cells in the G2/M phase decreases, and the expression levels of LC3-II/LC3-I increases and the expression level of p62 protein decreases, suggesting that inhibiting lncRNA MALAT-1 may strengthen the sensitivity of DLBCL to chemotherapy via enhancing autophagy. In this study, it was proved that lncRNA-SMAD5-AS1 was significantly down-regulated in DLBCL tissues and cell lines, and it could inhibit the tumor proliferation in vivo and in vitro, indicating that it may be a potential marker for diagnosis and treatment.

A variety of biological functions of lncRNA are dependent on the unique intracellular localization to a large extent. According to the ceRNA hypothesis proposed by researchers at the Harvard University in 2011, there is an interactive mode between miRNA and mRNA, and different types of RNA molecules (including mRNA and lncRNA) can regulate one another via the competitive inhibition on miRNA as long as there are common miRNA response elements (MRE). In other words, the concentration of target miRNA will increase when mRNA is down-regulated, thus down-regulating those mRNAs with the same MRE^30^. LncRNA expressed in the cytoplasm can regulate its stability and transcription through trapping miRNAs, thereby affecting the related signaling pathways^31^. In this study, the bioinformatic prediction, luciferase reporter gene assay, RIPA, and RNA pull-down assay were adopted to confirm that lncRNA-SMAD5-AS1 directly bind to miR-135b-5p and negatively regulated its activity. It has been proved in previous studies that miR-135b-5p plays an important role in various cancers, which can promote the occurrence and development of cancer and exert a cancer-promoting effect^32–37^. Besides, some studies also argued that miR-135b can inhibit the tumor development and reverse the drug resistance^38–40^. This study provides a certain basis for its cancer-promoting effect, and miR-135b-5p can reverse the anti-proliferation effect caused by the up-regulation of SMAD5-AS1. Xue et al.^41^ studied and argued that miR-135b-5p can be specifically adsorbed by lncRNA GAS5 in NSCLC, thus reducing the tumorigenicity and raising the sensitivity to radiotherapy. The similar ceRNA regulatory mechanisms in other cancers have also confirmed the findings to some extent. Noticeably, not only SMAD5-AS1 affected the activity of miR-135b-5p, but miR-135b-5p may also modulate the expression of SMAD5-AS1. As shown in Fig. 5e, the expression of SMAD5-AS1 altered when cells transfected with miR-135b-5p mimic or inhibitor. Sponge interaction of lncRNA/miRNA did not change the detectability of lncRNA. We may deduce that the absorption of miR-135b-5p may also affect the degradation of SMAD5-AS1. It was a predominant process in SMAD5-AS1/miR-135-5p ceRNA mechanism, which needs to be further confirmed in the future.

The Wnt signaling pathway is a well-known signaling pathway in the occurrence and development of cancer, whose specific process is as follows: Wnt protein binds to the seven-span transmembrane receptor in the frizzled family on the cell membrane, which delivers the signals into cells, activates the disheveled protein in the cytoplasm, inactivates the glycogen synthase kinase-3β (GSK-3β), and ensures that β-catenin in the cytoplasm is not degraded by GSK-3β. After β-catenin accumulates in the cytoplasm, it will enter the nucleus and form the complex with the TCF, thus activating the transcription of downstream genes. β-Catenin is degraded via phosphorylation by the complex formed by GSK-3β, APC, and axin/conductin in the case of no Wnt protein action^42^. Therefore, β-catenin is a positive regulator for the Wnt pathway, while APC is an important negative regulator. The negative regulation of APC on Wnt pathway has been confirmed in some studies^43–45^. It has also been reported that miR-135b can affect the tumor metastasis via the Wnt pathway^33^. In this study, it was also found that SMAD5-AS1 can affect the activation of Wnt/β-catenin pathway and directly bind to miR-135b-5p. Based on the further studies, the APC gene is a direct target of miR-135b-5p, and inhibiting the miR-135b-5p expression can significantly reduce the activity of TOP/FOP, indicating that the Wnt/β-catenin pathway is inhibited and the co-transfection with shSMAD5-AS1/SMAD5-AS1-overexpressed plasmid and miR-135b-5p inhibitor/mimic can increase or decrease the activity of TOP/FOP. The above results demonstrate that the SMAD5-AS1/miR-135b-5p axis can affect the activation level of the classical Wnt/β-catenin pathway via the specific regulation on the APC expression.

In conclusion, the results of this study indicate that the expression of lncRNA-SMAD5-AS1 declines in DLBCL, and its anti-tumor proliferation biological function in DLBCL was confirmed via the in vivo and in vitro experiments. In addition, its possible regulatory mechanism was found: it directly binds to miR-135b-5p to reduce its expression level, thus up-regulating the APC expression and lowering the activation level of the Wnt/β-catenin pathway. LncRNA-SMAD5-AS1 may be a new biomarker and a potential therapeutic target for DLBCL, but there are few studies on it so far, and its function and regulatory mechanism in the tumors remain unclear. At the same time, the function of lncRNA-SMAD5-AS1/miR-135b-5p axis in DLBCL needs further confirmation through large-sample clinical research, and it will also be the focus in the future.

## Data Availability

The datasets used and analyzed during the current study are available from the corresponding author on reasonable request.
